# HSP70 Inhibition Blocks Adaptive Resistance and Synergizes with MEK Inhibition for the Treatment of *NRAS*-Mutant Melanoma

**DOI:** 10.1158/2767-9764.CRC-21-0033

**Published:** 2021-10-13

**Authors:** Joshua L.D. Parris, Thibaut Barnoud, Julia I.-Ju Leu, Jessica C. Leung, Weili Ma, Nicole A. Kirven, Adi Naryana Reddy Poli, Andrew V. Kossenkov, Qin Liu, Joseph M. Salvino, Donna L. George, Ashani T. Weeraratna, Qing Chen, Maureen E. Murphy

**Affiliations:** 1 Program(s) in Molecular and Cellular Oncogenesis, The Wistar Institute, Philadelphia, Pennsylvania.; 2 Graduate Group in Cell and Molecular Biology, University of Pennsylvania Perelman School of Medicine, Philadelphia, Pennsylvania.; 3 Department of Genetics, University of Pennsylvania Perelman School of Medicine, Philadelphia, Pennsylvania.; 4 Immunology, Microenvironment and Metastasis, The Wistar Institute, Philadelphia, Pennsylvania.; 5 Gene Expression and Regulation, The Wistar Institute, Philadelphia, Pennsylvania.; 6 Department of Biochemistry and Molecular Biology, Johns Hopkins University, Baltimore, Maryland 21205.

## Abstract

*NRAS*-mutant melanoma is currently a challenge to treat. This is due to an absence of inhibitors directed against mutant *NRAS*, along with adaptive and acquired resistance of this tumor type to inhibitors in the MAPK pathway. Inhibitors to MEK have shown some promise for *NRAS*-mutant melanoma. In this work, we explored the use of MEK inhibitors for *NRAS*-mutant melanoma. At the same time, we investigated the impact of the brain microenvironment, specifically astrocytes, on the response of a melanoma brain metastatic cell line to MEK inhibition. These parallel avenues led to the surprising finding that astrocytes enhance the sensitivity of melanoma tumors to MEK inhibitors (MEKi). We show that MEKi cause an upregulation of the transcriptional regulator ID3, which confers resistance. This upregulation of ID3 is blocked by conditioned media from astrocytes. We show that silencing ID3 enhances the sensitivity of melanoma to MEKi, thus mimicking the effect of the brain microenvironment. Moreover, we report that ID3 is a client protein of the chaperone HSP70, and that HSP70 inhibition causes ID3 to misfold and accumulate in a detergent-insoluble fraction in cells. We show that HSP70 inhibitors synergize with MEKi against *NRAS*-mutant melanoma, and that this combination significantly enhances the survival of mice in two different models of *NRAS*-mutant melanoma. These studies highlight ID3 as a mediator of adaptive resistance, and support the combined use of MEK and HSP70 inhibitors for the therapy of *NRAS*-mutant melanoma.

**Significance:**

MEKi are currently used for *NRAS*-mutant melanoma, but have shown modest efficacy as single agents. This research shows a synergistic effect of combining HSP70 inhibitors with MEKi for the treatment of *NRAS* mutant melanoma.

## Introduction

Approximately 50% of melanoma tumors have a mutation in the serine-threonine kinase v-raf murine sarcoma viral oncogene homolog B1 (BRAF; refs. 1, 2). Mutant BRAF hyperactivates the RAS–RAF–MEK–MAPK pathway to support tumorigenesis by inducing cell-cycle dysregulation, activating prosurvival pathways, and promoting cellular proliferation (3, 4). The BRAF inhibitors (BRAFi) vemurafenib and dabrafenib were approved for use as single agents in patients with *BRAF*-mutant melanoma (5, 6). However, resistance often occurs due to reactivation of the RAS–RAF–MAPK pathway through multiple mechanisms (7, 8). Inhibitors of the MEK are frequently used in combination with BRAFi, markedly improving the survival of patients with *BRAF*-mutant tumors, and this combination is FDA approved for use in *BRAF^V600E^*-mutated melanoma (9–11). The second largest class of melanoma tumors contain mutations in the neuroblastoma RAS viral (v-ras) oncogene homolog (*NRAS*) gene. *NRAS* mutations are found in approximately 20% of patients with cutaneous melanoma (1, 2). Like *BRAF* mutations, mutations in *NRAS* lead to enhanced activation of the MAPK pathway. Clinical trials assessing the efficacy of MEKi PD0325901 (PD901), binimetinib and trametinib as single agents have produced modest results in patients with *NRAS*-mutant melanoma (1, 12), highlighting the need for agents that can improve the durability of this response. Furthermore, patients with activating mutations in *NRAS* have a poorer prognosis, are refractory to BRAF inhibition, and exhibit a greater incidence of brain metastases than patients with either *BRAF*-mutant or *BRAF/NRAS* wild-type melanoma (1, 13).

Melanoma brain metastases (MBM) occur in 40% to 60% of patients with metastatic melanoma. Patients with MBMs have an overall survival of less than 6 months from the time of diagnosis (14). One variable leading to the poor prognosis of MBMs is acquired resistance to chemotherapy; part of this resistance is hypothesized to occur through MBM interactions with astrocytes (15, 16). However, the impact of the brain microenvironment on the efficacy of inhibitors in the MAPK pathway has not been investigated. Astrocytes are the most abundant cell type in the central nervous system (CNS) and are important for maintaining homeostasis of the brain microenvironment. Upon insult, astrocytes become reactive, and normally serve to protect neurons from injury-induced apoptosis (17, 18). Reactive astrocytes have been shown to protect MBMs, and other brain metastases, via multiple pathways: through upregulation of survival genes (16, 19), sequestration of intracellular calcium (20), and secretion of miRNAs and prosurvival factors (21–23). In all cases tested, coculture of tumor cells with astrocytes, or incubation of tumor cells in conditioned media from astrocytes, has led to improved tumor survival (16, 20, 24). Here we report the surprising result that astrocyte conditioned media (ACM), or astrocyte coculture, increases the efficacy of the MEK inhibitor PD901 against the primary WM4265.2 BrM1 (WM4265.2) brain metastatic cell line (25). We show that MEK inhibitors cause upregulation of inhibitor of differentiation protein 3 (ID3), but not when melanomas are cultured in ACM. We show that silencing ID3 increases melanoma sensitivity to MEK inhibitors. We identify ID3 as a novel client protein of HSP70, and show that the HSP70 inhibitor AP-4–139B synergizes with MEK inhibitors against *NRAS*-mutant melanoma *in vivo*. This novel combination is a promising therapeutic avenue for *NRAS*-mutant melanoma.

## Materials and Methods

### Cell Lines

Human astrocytes and astrocyte media (AM) were obtained from ScienCell (1800, 1801). The WM4265.2 BrM1 (WM4265.2), WM983B, and 1205Lu cells lines were provided by Qing Chen and Meenhard Herlyn (The Wistar Institute, Philadelphia, PA). The WM4265.2 cell line constitutively express GFP and luciferase. M93–047 cells were provided by Jessie Villanueva (The Wistar Institute, Philadelphia, PA). MaNRAS1014 cells (26) were obtained from Andrew Aplin (Thomas Jefferson University Cancer Center, Philadelphia, PA) and Lionel Larue (Institute Curie, Paris, France). WM4265.2 cells were grown in DMEM (Corning: 10–013-CM) supplemented with 10% FBS (Hyclone, GE Healthcare Life Sciences), 1% penicillin/streptomycin (Gibco: 15140122), and 1% Glutamax (Gibco: 35050–061). 1205Lu and WM983B were grown in Tu 2% consisting of MCDB153 (Sigma-Aldrich: M7403–1L), 20% Leibovitz L-15 Medium (Gibco: 11415–064), 2% FBS, 1% penicillin/streptomycin, and 1.68 mmol/L CaCl_2_. MaNRAS1014 cells were grown in Ham F12 Nutrient Mix (Gibco: 11765–054), 10% FBS, 1% penicillin/streptomycin, and supplemented with 10 nmol/L TPA. M93–047 cells were grown in RPMI1640 (Corning: 10–040-CM), 5% FBS, and 1% penicillin/streptomycin. Cell lines were incubated in a 5% CO_2_ humidified incubator at 37°C. All cell lines were used within six months of obtaining them from the sources described; cell line identity was confirmed using short tandem repeat profiling, and cells were tested for *Mycoplasma* every six months by the MycoAlert assay (University of Pennsylvania Cell Center, Philadelphia, PA).

### Antibodies, Reagents, and Western Blot Analysis

The following antibodies were used: ID3 (9837S), cleaved caspase 3 (9961S), cleaved Lamin A (2035S), HSP90 (4887S), EGFR (4267S), AKT (9272S), GAPDH (2218S), p44/42 (4695S), p-p44/42 (9101S), V5-Tag (13202; Cell Signaling Technology), HSP70 (C92F3A-5; Enzo Life Sciences). PD0325901 (S1036) and trametinib (S2673) were purchased from Selleckchem. U1866a (U3633) was purchased from Sigma-Aldrich. AP-4–139B was generated in the Molecular Screening Facility at The Wistar Institute and confirmed by nuclear magnetic resonance (NMR). For *in vitro* studies, PD0325901, trametinib, and AP-4–139B were dissolved in DMSO. The following siRNAs were used: Accell Human ID3 SMARTpool (E-009905–01–0020), ON-TARGET plus Human SREBF1 SMARTpool (L-006891–00–0020), Accell Human SREBF2 SMARTpool (E-009549–00–010), Accell Non-targeting Pool (D-001910–10–20, Dharmacon). For Western blot analyses, 25–100 μg of protein was run over SDS-PAGE gels using 10% NuPAGE Bis-Tris precast gels (Life Technologies) and were transferred onto polyvinylidene difluoride (PVDF) membranes (IPVH0010, pore size: 0.45 mm; Millipore Sigma). Following transfer, membranes were blocked using ether 5% nonfat dry milk or 5% BSA (Sigma Aldrich: A9647) for 1 hour at room temperature. Membranes were probed with indicated antibodies. Rabbit or mouse secondary antibodies conjugated to horseradish peroxidase (Jackson Immunochemicals) were used at 1:10,000 dilution and treated with Pierce ECL Western Blotting Substrate (Thermo Scientific: 32106), SuperSignal West Femto Maximum Sensitivity Substrate (Thermo Scientific: 34095), or Amersham ECL Prime Western Blotting Detection Reagents (GE Healthcare: RPN2232) for 3–5 minutes. Protein levels were detected using autoradiography, and densitometry analysis of proteins was conducted using Image J software (NIH, Rockville, MD).

### Animal Studies

All studies were carried out in accordance with the recommendations in the Guide for the Care and Use of Laboratory Animals of the NIH (Bethesda, MD). All protocols were approved by The Wistar Institute Institutional Animal Care and Use Committee (IACUC). Mice were housed in plastic cages with *ad libitum* diet and maintained with a 12-hour dark/12-hour light cycle at 22°C. For xenograft studies, 2.5 × 10^6^ MaNRAS1014 or 1 × 10^6^ M93–047 cells were injected subcutaneously into the right flanks of 6- to 8-week-old male NSG (NOD.Cg-Prkdcscikd II2rgtm1Wjl/Szj) mice. For drug treatments, mice were given 2 mg/kg/day PD0325901 (SelleckChem: S1036; 2 mg/kg/day in 0.2% Tween 80, 0.5% methylcellulose, 5% DMSO) by oral gavage and AP-4–139B (synthesized by the Wistar Molecular Screening Facility, validated by NMR; 10 mg/kg in 2% DMSO in 0.9% NaCl solution) every other day by intraperitoneal injection. Tumor volumes were measured using digital calipers, and tumor volume was calculated using the following formula: volume = (length × width^2^) × 0.5. Body weight was measured every other day. All mice were monitored daily for signs of pain or distress.

### ACM and Astrocyte Coculture

1 × 10^6^ primary human astrocytes (ScienCell: 1800) were plated in T-75 tissue culture flasks in AM (ScienCell: 1801) and incubated for 48 hours until cells were approximately 90% confluent. Conditioned media was collected from four passages (p0–p4) from two different batches of astrocytes, spun at 2,000 rpm to remove cells and debris, combined and frozen at −80°C until needed. WM4265.2, WM983B, and 1205Lu cell lines were acclimated to AM for four passages and incubated for 48 hours in ACM for 48 hours prior to Western Blot, qPCR and cell viability assays. For astrocyte coculture experiments, astrocytes (p4–p7) were cultured in a 1:1 ratio with WM4265.2 for 24 hours. WM4265.2 monoculture and astrocyte cocultures were treated with PD0325901 for 24 hours and cell viability was determined.

### Luciferase, Annexin V, Cell-cycle Analysis

WM4265.2 mono- and astrocyte cocultures were seeded in 100 μL in a black 96-well plate (CytoOne: CC2682–769) and incubated for 24 hours at 37°C in a 5% CO_2_ humidified chamber. Mono- and cocultures were treated with PD0325901 for 24 hours (final volume 200 μL) and luciferase activity was assessed. 20 μL of 0.375 mg/mL d-Luciferin was added to each sample, and luminescence was read immediately using an IVIS machine. Luminescence was normalized to untreated controls. For Annexin V measurements, cells plus media were collected and pelleted at 1,200 rpm and washed in cold 1× PBS. Pellets were resuspended in 100 μL of 1× Annexin binding buffer (Invitrogen: V13246) and stained with 5 μL Annexin V, R-phycoerythrin (Invitrogen: A35111) in the dark for 15 minutes at room temperature. Four-hundred microliters of 3 μmol/L DAPI in 1× annexin binding buffer was added to each sample. Annexin V staining of GFP^+^ WM4265.2 was analyzed via flow cytometry. For cell-cycle analysis in WM4265.2 mono- and cocultured cells, media and cells were collected and pelleted at 1,000 rpm. Single-cell suspensions were obtained via pipetting 750 μL 1× PBS. 16% PFA (250 μL) was added directly to single-cell suspension (final concentration 4%) and incubated at room temperature for 15 minutes to fix. This fixation protocol was used to retain GFP signal in the WM4265.2 cells. After fixation, cells were centrifuged at 500 × *g* for 5 minutes, PFA was decanted and pellet was washed once with PBS. Cells were resuspended in 500 μL of 1× PBS and pipetted to obtain a single-cell suspension. Three milliliters of cold 70% ethanol was added directly to each tube and incubated overnight at 4°C. Cells were centrifuged at 500 × *g* for 8 minutes to pellet and ethanol was carefully decanted. Cells were washed two times with cold 1× PBS and resuspended in 500 μL FxCycle PI/RNase Staining Solution (Invitrogen: F10797). Samples were incubated for 20–30 minutes at room temperature in the dark and cell cycle was analyzed via flow cytometry. For all other cell-cycle analyses, cell pellets were resuspended in 500 μL of 1× PBS to obtain a single-cell suspension. Three milliliters of cold 70% ethanol was added directly to each tube and cells were fixed for 30 minutes.

### Cell Viability and Synergy Assays

For ACM studies, WM4265.2 and WM983B were incubated in either AM or ACM for 48 hours. 2 × 10^4^ WM4265.2 and WM983B were plated in a flat bottom 96-well plate (Corning) in either 50 μL AM or ACM and incubated overnight at 37°C in a 5% CO_2_ humidified chamber. Cisplatin, doxorubicin, and PD0325901 were prepared via serial dilution from 200 μmol/L to 0.002 μmol/L and 50 μL was added to each well for 72 hours (final concentration 100 μmol/L to 0.001 μmol/L). Ten microliters (10% volume) AlamarBlue (Invitrogen: DAL1025) was added to each well and incubated for up to 4 hours at 37°C in 5% CO_2_ humidified chamber. Cell viability was determined by fluorescence at 560/590 using the Synergy HT plate reader (BioTek). For synergy assays, WM4265.2 and MaNRAS1014 cells were plated at 500 cells per well in white 384-well plates in 20 μL of complete media using the Biotek Microflo and incubated overnight at 37°C in a 5% CO_2_ humidified chamber. PD0325901, trametinib, and AP-4–139B, were serially diluted in 100% DMSO at 1,000× final concentration. Titrated compounds were then diluted 1:250 into complete media and 10 μL were then added to the appropriate wells. Once both compounds were added, the final DMSO concentration in the media was 0.2% in 40 μL of complete media. The cells were treated with the appropriate combination of compounds for 72 hours at 37°C in a 5% CO_2_ humidified chamber. After 72 hours, 20 μL of CellTiterGlo was added to the plates and luminescence was measured using the Envision. Data were normalized to % toxicity where 0% toxicity is the counts in the absence of drug, and 100% toxicity is the counts in the presence of 10 μmol/L bortezomib. Nonlinear regression fits of the data were performed using XLfit software (IDBS). Synergy was determined using an interaction index calculated using a dose–response surface model based on the Bliss independence principle (27, 28). For combinations when the interaction index and upper limit of its 95% confidence interval < 1, the combination effect of the two drugs was considered significantly synergistic.

### Colony-Forming Assay

For colony formation assays, 1 × 10^4^ MaNRAS1014 or WM4265.2 cells were plated in a 6-well plate. Once cells were adherent, cells were treated once with AP-4–139B, PD0325901, and trametinib as single agents, or in combination as indicated for 7 days. After 7 days, cells were fixed to the plates using 10% formalin, and stained with 0.5% Crystal Violet (Sigma Aldrich: C3886–100G) for 1 hour. The percentage of Crystal Violet staining relative to the total area of each well was compared between treatment groups.

### Proximity Ligation Assay

Cells were grown on Lab-Tek II 8-well chamber slides and fixed with 4% paraformaldehyde (Electron Microscopy Sciences: 15710), followed by permeabilization with 0.25% Triton X-100 (Millipore Sigma: 1132481001). Protein–protein interactions were assessed using the PLA Duolink In Situ Starter Kit (Sigma-Aldrich: DUO92101) according to the manufacturer's protocol, using the following primary antibodies: ID3 1:200 and HSP70 1:50. Slides were mounted with media containing DAPI and images were captured on a Leica TSC SP5 microscope. ImageJ software (NIH, Rockville, MD) was used to quantify proximity ligation analyses (PLA) signals.

### Soluble Insoluble Fractionation

Proteins were extracted from cultured cells using Lysis Buffer (50 mmol/L Tris-HCl, pH 7.5; 150 mmol/L NaCl; 2 mmol/L EDTA; 1% IGEPAL CA-630; and 0.5% Triton X-100) supplemented with protease inhibitors at 4°C. Cell lysates were spun at 11,000 × *g* for 30 minutes at 4°C and the supernatants contained the detergent-soluble fraction. The pellets containing the detergent-insoluble fractions were resuspended using the Lysis Buffer. Both the detergent-soluble and -insoluble protein samples were size fractionated on Novex 4%–20% Tris-Glycine Mini Protein Gels (Thermo Fisher Scientific: XP04200BOX) and transferred overnight onto Immuno-Blot PVDF membranes at 4°C. The membranes were blocked for 30 minutes at room temperature using 3% Blotting-Grade Blocker in 1× PBST, and incubated with indicated antibodies overnight with rotation/nutation at 4°C. The next day, the membranes were washed in 1× PBST, incubated with indicated secondary antibodies for 2 hours at room temperature, and proteins were detected using ECL Western blotting detection reagents.

### Co-immunoprecipitation

Following overnight seeding of M93–047 cells (> 75% confluent), the cells were harvested and centrifuged at 2,000 rpm for 5 minutes at 4°C. Pellets were lysed in 300 μL of Pierce IP Lysis Buffer (Thermo Fisher: 87787) with 1x Halt Protease Phosphatase Inhibitor Cocktail (Thermo Fisher: 78440) and incubated on ice for 5 minutes. Cellular lysates were spun at 13,000 × *g* for 10 minutes at 4°C. Protein extracts (2 mg per reaction) were incubated with HSP70/HSP72 antibody for 1 hour at 4°C with rotation. HSP70-immunocomplexes were captured using Protein G agarose beads (Cell Signaling Technology: 3748) and rotated for 30 minutes at 4°C. Equal volumes of 2× Laemmli Sample Buffer were added to each reaction, and samples were boiled for 10 minutes at 95°C. HSP70-associated proteins were analyzed by Western blot, using HSP70/HSP72 and ID3 antibodies.

### RNA-sequencing and qRT-PCR

Following treatments, cells were harvested and lysed on QIAshredder columns (Qiagen: 79656). Total RNA was extracted from cells using the Qiagen RNeasy Mini Kit (Qiagen: 74106) according to the manufacturer's protocol. RNA quantity was determined using the Qubit 2.0 Fluorometer (ThermoFisher Scientific) and the quality was validated using the TapeStation RNA ScreenTape (Agilent). Five-hundred nanograms of DNAse I treated, total RNA was used to prepare library for Illumina Sequencing using the Quant-Seq 3′mRNA-Seq Library Preparation Kit (Lexogen). Library quantity was determined using qPCR (KAPA Biosystems). Overall library size was determined using the Agilent TapeStation and the DNA High Sensitivity D5000 ScreenTape (Agilent). Equimolar amounts of each sample library were pooled, denatured and high-output, single-read, 75-bp cycle, next generation sequencing was done on a NextSeq 500 (Illumina).RNA-sequencing (RNA-seq) data was aligned using bowtie2 (29) against hg38 version of the human genome and RSEM v1.2.12 software (30) was used to estimate raw read counts for each gene using Ensemble v84 transcriptome information. DESeq2 (31) was used to estimate significance of differential expression between sample groups. Genes differentially expressed between conditions at nominal *P* < 0.05 were analyzed using QIAGEN's Ingenuity Pathway Analysis software (IPA, QIAGEN, www.qiagen.com/ingenuity) using “Canonical Pathway” option. The data were uploaded to NCBI GEO database and are available under accession number GSE179235. For qRT-PCR analysis, RNA quality and concentration was determined via nano-drop. Equal amounts of isolated RNA were converted to cDNA via reverse transcription using a High Capacity cDNA Reverse Transcription Kit (Applied Biosystems: 4368814). qPCR was performed using Brilliant III Ultra-Fast SYBR QPCR Master Mix (Agilent Technologies: 600882) on a Stratagene Mx3005P machine (Agilent Technologies). Data analysis of relative transcript quantity was performed using MxPro program (Stratagene) and GraphPad Prism. RNA expression for each transcript was normalized to TBP or GAPDH.

### Statistical Analysis

Unless otherwise stated, all experiments were carried out with a minimum of three biological replicates (*n* = 3). All mouse experiments had 7–12 animals per experimental group. Linear mixed models were used to analyze longitudinal tumor growth measures. The log-rank test was used to analyze time to tumor growth data and survival data. The Student *t* test or Wilcoxon rank sum test were used for analyzing continuous variables. For *in vitro* studies, the two-tailed unpaired Student *t* test was performed for two-group comparisons. One-way ANOVA with *post hoc* Holm-Šídák multiple comparisons test was used for multigroup comparisons. For drug combination effect analysis with *in vitro* data, Bliss independence models were applied, and interaction indexes were calculated to determine synergistic effect as described. All *in vitro* data are reported as the mean ± SD unless stated otherwise, and all *in vivo* data are reported as the mean ± SE. Statistical analyses were performed using GraphPad Prism 9.1.0 (GraphPad Software) and R 4.0.3. *P* values are as indicated: *, *P* < 0.05; **, *P* < 0.01; ***, *P* < 0.001; n.s., not statistically significant.

### Data Availability Statement

RNA-seq data were deposited to NCBI GEO database and is available under accession number GSE179235. For additional materials and methods, see the online Supplementary Materials and Methods.

## Results

### Astrocytes Increase the Sensitivity of Melanoma Cells to MEKi

**FIGURE 1:**
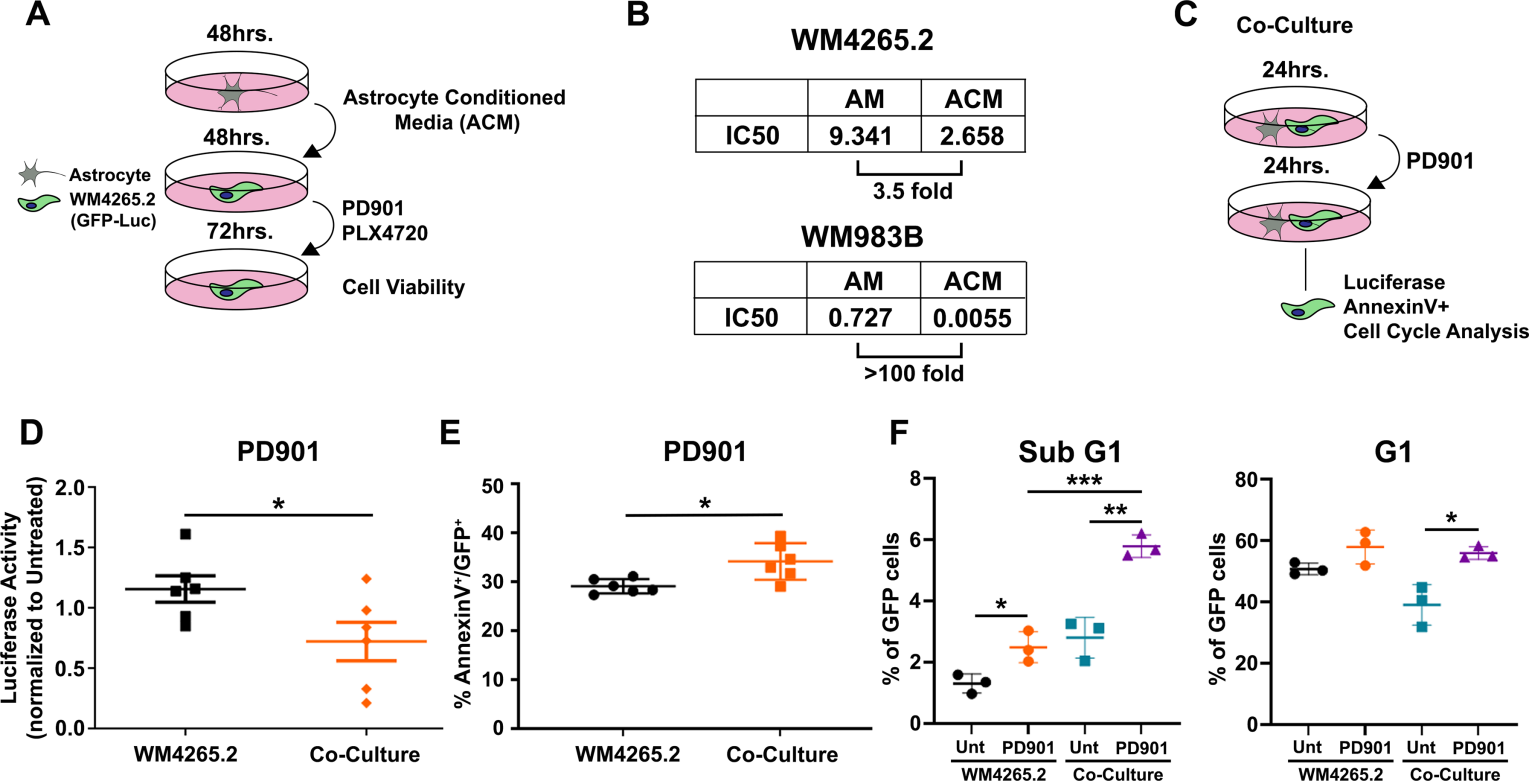
ACM or coculture increases melanoma sensitivity to MEK inhibition. **A,** 1 × 10^6^ primary human astrocytes were cultured for 48 hours, after which conditioned media was collected, combined, and frozen until use. Media was collected from four passages. WM4265.2 and WM983B cell lines were incubated in AM or ACM for 48 hours and treated with cisplatin, doxorubicin, PD0325901 (PD901), and PLX4720 for 72 hours. Cell viability was determined using AlamarBlue assays. **B,** IC_50_ for PD901 of WM4265.2 and WM983B cell lines incubated in AM or ACM. Fold decrease in IC_50_ value in ACM is depicted below and represent *n* = 6. **C,** Experimental design for astrocyte plus melanoma coculture analysis: cells were cocultured at 1:1 ratios for 24 hours. **D,** Luciferase activity of WM4265.2 in monoculture and cocultures after 24-hour PD901 treatment. Data shown represent *n* = 6. *, *P* < 0.05; assessed by two-tailed Student *t* test. **E,** Flow cytometric analysis of Annexin V^+^/GFP^+^ WM4265.2 cells in mono- and astrocyte cocultures treated with PD901. Data shown represent *n* = 6. *, *P* < 0.05; assessed by two-tailed Student *t* test. **F,** Flow cytometric analysis of GFP^+^ WM4265.2 cells grown as mono- or cocultures with astrocytes for 24 hours. Sub-G_1_ (apoptotic) and G_1_ phase cells are depicted from *n* = 6. ***, *P* < 0.001; **, *P* < 0.01; *, *P* < 0.05; assessed by one-way ANOVA. All PD901 treatments were 10 μmol/L.

Targeted therapy using BRAFi and MEKi has shown significant impact on melanoma survival. However, this combination cannot be used for *NRAS*-mutant melanoma, and currently *NRAS*-mutant melanoma is treated with immunotherapy or MEK inhibitors as first-line therapy. Recent studies suggest that the efficacy of melanoma therapy can be markedly affected by the tumor microenvironment (32). In particular, astrocytes in the brain microenvironment promote the survival of melanoma and other brain metastases (16, 19, 20). To date, however, the impact of astrocytes on the response of melanoma to inhibitors in the MAPK pathway has not been established. To begin to address this issue, we collected conditioned media from cultures of primary human astrocytes and assessed the IC_50_ for several drugs in melanoma cultured in AM or ACM (see schematic, Fig. 1A). We first tested the human melanoma brain metastatic cell line WM4265.2, which is derived from a patient-derived xenograft tumor and has a mutation in *NRAS* (25). We first confirmed previous reports (20) that astrocytes conferred resistance of WM4265.2 cells to the genotoxic agents cisplatin and doxorubicin (Supplementary Fig. S1A). Surprisingly however, we found that ACM-rendered WM4265.2 cells more sensitive to the MEKi PD901 (Fig. 1B). This increased sensitivity was also evident in the WM983B melanoma line, which has a *BRAF* mutation (Fig. 1B). ACM also rendered WM983B cells more sensitive to the BRAFi PLX4720 (Supplementary Fig. S1B).

We next sought to corroborate these findings in melanoma cells cocultured with astrocytes (see schematic Fig. 1C). The WM4265.2 cell line constitutively expresses luciferase and GFP; we cocultured these cells with an equal number of primary human astrocytes and treated with the MEKi PD901 for 24 hours. We next assessed luciferase activity as a surrogate for cell viability, and also assayed apoptosis of GFP-positive cells using two assays (Annexin V staining and sub-G_1_ content via flow cytometry). Consistent with our conditioned media experiments, we found that coculture of WM4265.2 with astrocytes led to decreased luciferase activity (increased sensitivity) compared with melanoma cells cultured alone (Fig. 1D). We also found significantly increased Annexin V^+^ staining (Fig. 1E), and increased sub-G_1_ content (Fig. 1F) in GFP-positive cocultured melanoma cells. We noted that PD901 treatment caused an accumulation of cells in G1, indicative of growth arrest, and that coculture with astrocytes appeared to enhance this response (Fig. 1F; Supplementary Fig. S1C). Together, these data suggest that astrocytes may increase the sensitivity of melanoma to MEK inhibition by enhancing apoptosis, but also potentially by increasing growth arrest.

### The Transcriptional Regulator ID3 Is Induced By PD901 Treatment; This Upregulation Is Lost when Melanoma Is Cultured in ACM

**FIGURE 2:**
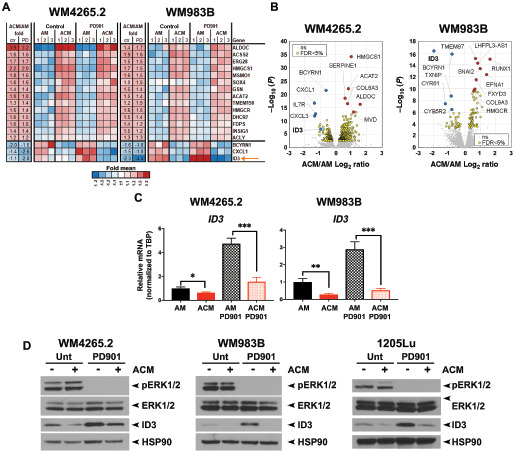
ACM prevents the upregulation of ID3 induced by PD901. **A,** Expression heatmaps of genes which by RNA-seq analysis were found to be commonly affected in WM4265.2 or WM983B cells incubated in the presence versus absence of ACM and PD901. Cells were incubated in ACM for 48 hours and PD901 (10 μmol/L) was added for 8 hours. **B,** Volcano plot highlighting the top up- and downregulated genes in WM4265.2 and WM983B cell lines treated with PD901 in the presence of AM or ACM. Significance of gene expression changes was defined using FDR threshold of 5%. **C,** qRT-PCR analysis of RNA levels of *ID3*, normalized to TBP, using independent samples of WM4265.2 and WM983B. Values shown are mean ± SD of *n* = 3. ***, *P* < 0.001; **, *P* < 0.01; *, *P* < 0.05 as per two-tailed Student *t* test. **D,** Western blot, probed with indicated antibodies, of lysates from WM4265.2, WM983B, and 1205Lu cell lines incubated in AM or ACM, and treated with PD901 (10 μmol/L) for 8 hours.

To determine the mechanism where astrocytes and ACM increase melanoma sensitivity to PD901, we performed RNA-seq analysis on the WM4265.2 and WM983B cell lines cultured in the presence or absence of ACM, in the presence or absence of PD901 (Fig. 2A and B). Differential gene expression and IPA revealed a significant enrichment of genes involved in the sterol biosynthesis pathway in both WM4265.2 and WM983B cell lines exposed to ACM (Fig. 2A and B; Supplementary Fig. S2A–S2C) suggesting that the master regulator, stable regulatory-element binding protein (SREBP1/2), might be involved. However, silencing of the gene encoding SREBP (*SREBF1)* in WM4265.2 and WM983B cells had opposing effects on cell viability following treatment with PD901 (Supplementary Fig. S2D), so we did not pursue this target further. Further inspection of the RNA-seq data revealed that the expression of ID3 was upregulated by PD901, but this was abrogated when melanomas were cultured in ACM (Fig. 2A, arrow). ID3 is a transcriptional regulator implicated in the resistance of melanoma to BRAFi (33). The upregulation of ID3 following PD901 treatment, and inhibition of this by ACM, was confirmed in several melanoma lines (WM4265.2, WM983B, and 1205Lu) at the RNA (qRT-qPCR, Fig. 2C) and protein levels (Fig. 2D). Interestingly, we found that the ability of ACM to prevent the upregulation of ID3 may rely on increased SREBP1/2 activity; in support of this, we found that the SREBP1/2 agonist U1866a decreased the expression of ID3 (Supplementary Fig. S2E).

**FIGURE 3:**
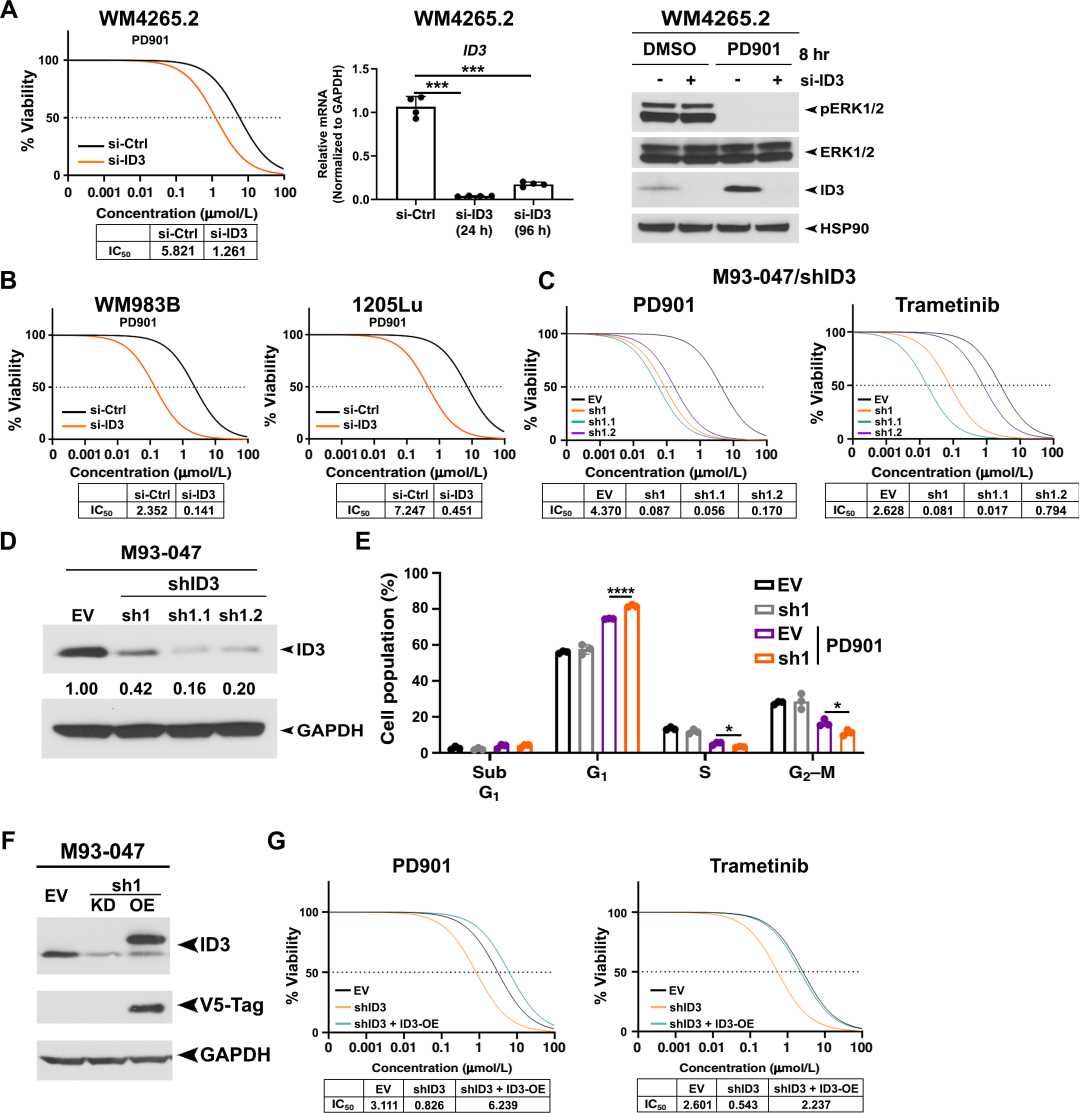
ID3 regulates sensitivity to MEKi. **A,** Left, IC_50_ analysis of WM4265.2 cells after incubation with siRNA targeting ID3 or nontargeting siRNA for 24 hours; cells were treated with PD901 for 72 hours and cell viability was measured using AlamarBlue assays. IC_50_ values shown are representative of 3-6 technical replicates. Middle panel, qRT-PCR of *ID3* level, normalized to GAPDH. Values shown are mean ± SD of *n* = 3. Western blot probed with indicated antibodies of lysates from WM4265.2 cells in the presence or absence of siRNA targeting *ID3* and PD901 (10 μmol/L). **B,** IC_50_ analysis of WM983B and 1205Lu cells incubated in nontargeting siRNA or siRNA targeting ID3 and treated with PD901 for 72 hours. IC_50_ values shown represent *n* = 3. **C,** IC_50_ analysis of M93–047 cells with stable knockdown of ID3 via shRNA infection. IC_50_ values shown represent *n* = 6. **D,** Western blot analysis, probed with indicated antibodies of lysates from M93–047 with pooled and two clones of shRNA targeting ID3 or empty vector (EV). **E,** Cell-cycle analysis of M93–047 cells with stable knockdown of ID3 or empty vector (EV) via flow cytometry. Values shown are the mean ± SD from *n* = 3. ***, *P* < 0.001; *, *P* < 0.05; assessed by two-tailed Student *t* test. **F,** Western blot analysis probed with indicated antibodies of lysates from M93–047 infected with empty vector (EV), shRNA for *ID3* (sh1) or shRNA for ID3 plus an *ID3* overexpression plasmid that is resistant to silencing (OE). **G,** IC_50_ analysis of the M93–047 clones in **F** treated with PD901 or trametinib. IC_50_ values shown represent *n* = 6.

We next sought to determine the impact of ID3 silencing and overexpression on the sensitivity of melanoma to MEK inhibition. Toward this end, we silenced ID3 with siRNA or shRNA and assessed the IC_50_ for MEK inhibitors. Silencing ID3 with siRNA (si-ID3) in WM4265.2, WM983B, and 1205Lu cells led to markedly increased sensitivity to PD901 in all three lines, to levels comparable to that achieved following incubation in ACM (Fig. 3A and B; Supplementary Fig. S3A and S3B). Stable expression of sh-ID3 in the *NRAS*-mutant melanoma M93–047 cell line also led to increased sensitivity to PD901 and to trametinib, another MEKi; this effect was recapitulated in pooled stably infected cell lines as well as two independent clones (Fig. 3C and D). Silencing of ID3 had no effect on the proliferation rate of M93–047 cells (Supplementary Fig. S3B) or on the ability of MEKi to block phospho-ERK (Supplementary Fig. S3C). Results using stable expression of two different short hairpins for ID3, and two different MEKi (PD901 and trametinib) were comparable (Supplementary Fig. S3D). To assess the downstream effect of ID3 silencing on the response to MEKi, we performed flow cytometry for cell cycle and cell death (sub-G_1_), trypan blue viability assays, and Western blots for cleaved lamin A, a marker of programmed cell death. Treatment with PD901 caused an increase in cells in the G_1_ phase of the cell cycle; this was enhanced in ID3-silenced cells (*P* < 0.001, Fig. 3E). We also noted decreased viability (Supplementary Fig. S3E) and increased apoptosis (Supplementary Fig. S3F and G). Overexpression of a nonsilenceable version of ID3 in these sh-ID3 cells attenuated the sensitivity to MEKi (Fig. 3F and G). These data suggest that ID3 is a mediator of melanoma sensitivity to MEK inhibition.

### ID3 Is a Client Protein of HSP70

**FIGURE 4:**
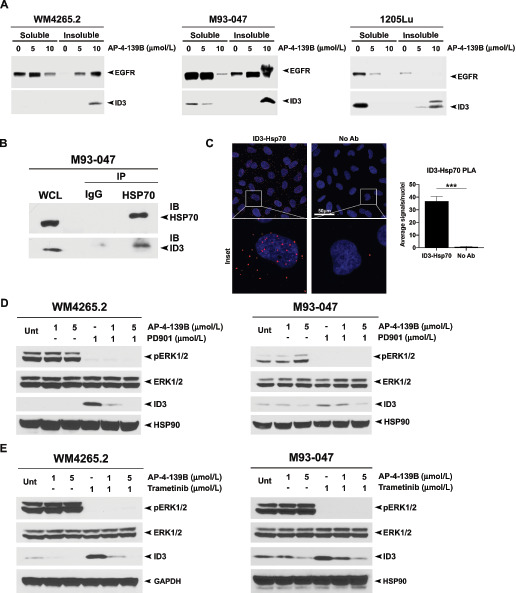
ID3 is a novel client protein of HSP70. **A,** Western blot probed with indicated antibodies of detergent-soluble and -insoluble lysates from WM4265.2, M93–047, and 1205Lu cell lines treated with 0, 5, and 10 μmol/L AP-4–139B for 24 hours. **B,** Lysates from M93–047 cells were immunoprecipitated with IgG or anti-HSP70 antibodies and probed for ID3. WCL, whole-cell lysate. **C,** PLA for HSP70–ID3 complexes in M93–047 cells. Individual HSP70-ID3 interactions are visualized by fluorescent signal (red) with nuclei counterstained with DAPI. Scale bar, 50 μm. Representative images are maximum intensity projects from z-stacks. Right, quantification of the HSP70–ID3 interactions measured as the average number of PLA signals per nuclei, from > 100 cells analyzed from random fields in each of two technical replicates. ***, *P* < 0.001, assessed by two-tailed Student *t* test. **D** and **E,** Western blot probed with indicated antibodies of lysates from WM4265.2 and M93–047 cell lines treated with the indicated concentrations of AP-4–139B, PD901, and/or trametinib for 24 hours.

The ability of ID3 to contribute to the resistance to MEKi in both *NRAS*-mutant (WM4265.2, M93–047) and *BRAF*-mutant (1205Lu, WM983B) melanomas suggested that it might be a good therapeutic target for melanoma. However, there are no inhibitors that target ID3. Because ID3 is an intrinsically unstable protein (34), we sought to determine whether it might interact with, and be regulated by, the HSP70. To do this, we first tested whether treatment with an HSP70 inhibitor would cause ID3 to localize in a detergent-insoluble fraction of the cell (due to misfolding). We performed a soluble–insoluble fractionation of WM4265.2, M93–047, and 1205Lu cell lines treated with our novel HSP70 inhibitor, AP-4–139B; whereas AP-4–139B is tagged with a triphenylphosphonium to increase distribution of the compound to the mitochondria, this inhibitor is broadly distributed, and it affects the solubility of client proteins in the cytosol and nucleus as well (35). Treatment of cells with increasing doses of AP-4–139B resulted in an increase of ID3 in the insoluble fraction in each of the cell lines tested, comparable with a known client, EGFR (Fig. 4A). Next, we assessed the ability of HSP70 to interact with ID3 using two assays, immunoprecipitation–Western and PLA. Immunoprecipitation with HSP70 antisera revealed ID3 in the immunoprecipitated complexes of M93–047 cells (Fig. 4B). Finally, PLA corroborated an interaction between HSP70 and ID3, which appeared to exist in both the nucleus and the cytosol (Fig. 4C). Given that treatment with PD901 causes increased ID3 expression in each of our melanoma cell lines tested, we next sought to determine whether HSP70 inhibition could block this. WM4265.2 and M93–047 cells were treated with the HSP70i AP-4–139B and PD901 as single agents, or in combination for 24 hours. Western blot analysis showed that combining AP-4–139B with PD901 prevented ID3 upregulation caused by MEKi in both cell lines (Fig. 4D). Similar findings were observed with the combination of trametinib and AP-4–139B (Fig. 4E). These data supported the testing of the combination of AP-4–139B with MEKi for melanoma therapy.

### Synergy between PD901 and AP-4–139B in the Treatment of Melanoma

**FIGURE 5:**
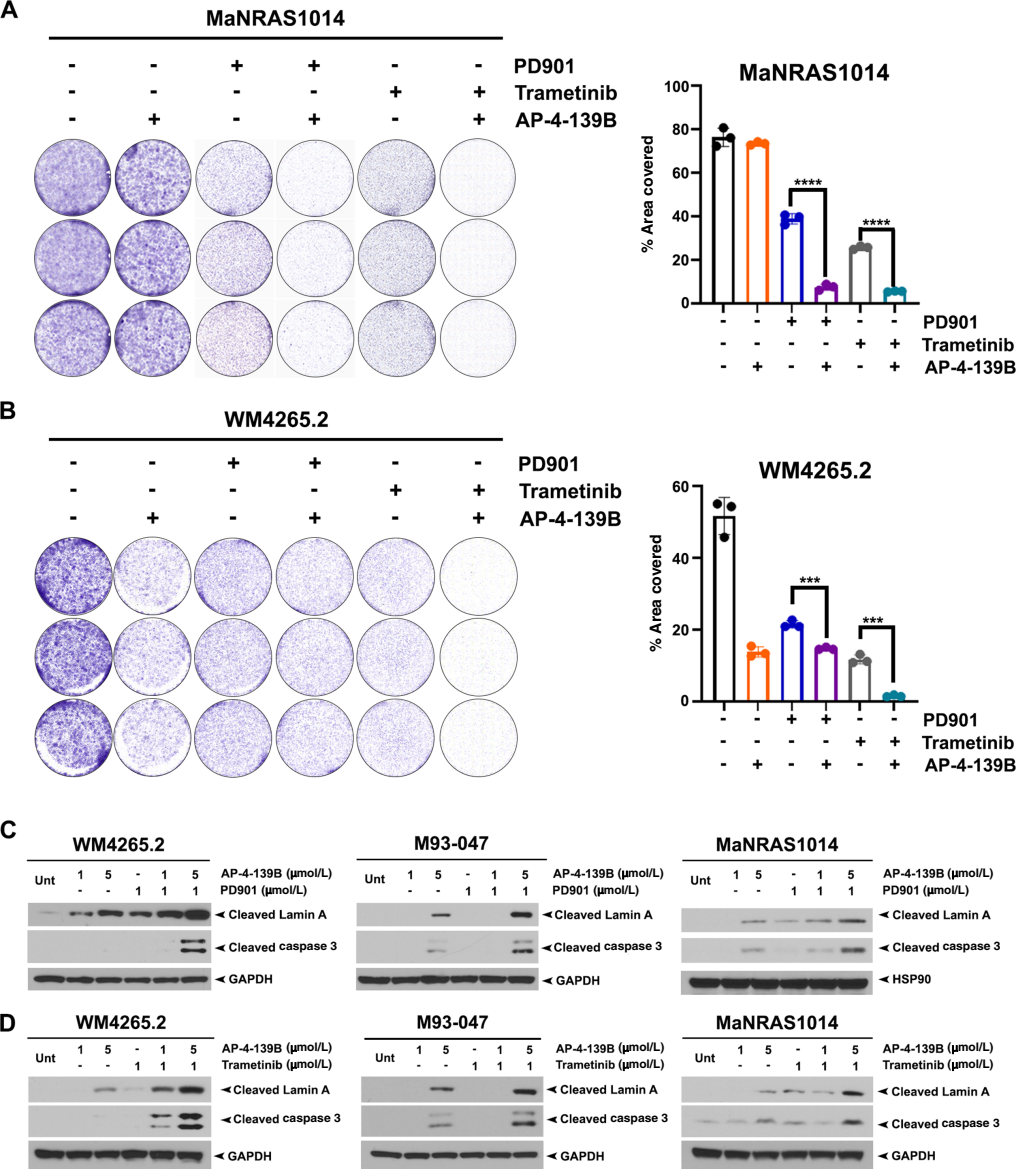
Combination PD901 and AP-4–139B synergizes in *NRAS*-mutant melanoma *in vitro*. **A** and **B,** 1 × 10^4^ MaNRAS1014 (**A**) and WM4265.2 (**B**) were treated with either 0.25 μmol/L PD901, 0.25 μmol/L trametinib, 1 μmol/L AP-4–139B, or combination of PD901 and AP-4–139B, and combination of trametinib and AP-4–139B. Seven days after treatment, cells were stained with 0.5% crystal violet. Quantification of crystal violet stain is shown on the right. Values shown represent *n* = 3 for each treatment group ± the SD. ***, *P* < 0.001; **, *P* < 0.01; *, *P* < 0.05; assessed by two-tailed Student *t* test. **C** and **D,** WM4265.2, M93–047, and MaNRAS1014 cells were treated with AP-4–139B, PD901, trametinib or combination at the indicated doses for 24 hours. Lysates were extracted and analyzed for cleaved lamin A and cleaved caspase 3 via Western blot analysis. GAPDH or HSP90 were used as loading controls.

We next sought to determine whether the combination of MEK and HSP70 inhibition was efficacious in the treatment of *NRAS*-mutant melanoma. Toward this end, we calculated the interaction index with a 95% confidence interval (95% CI) at six different doses of PD901 and AP-4–139B. Using this method, we observed a significant synergistic effect between PD901 and AP-4–139B in the WM4265.2 cell line (overall interaction index = 0.91; 95% CI, 0.86–0.98) and the murine MaNRAS1014 cell line (overall interaction index = 0.75; 95% CI, 0.69–0.83; Supplementary Fig. S4A–S4D). We found similar evidence for synergy between AP-4–139B and trametinib in both cell lines (Supplementary Fig. S4A–S4D). Flow cytometric cell-cycle analyses of treated cell lines revealed modest increases of the combination on G_1_ arrest, but more marked evidence for increased apoptosis (sub-G_1_ content, Supplementary Fig. S4E). These findings were extended to include clonogenic survival assays. We found that combining AP-4–139B with either PD901 or trametinib significantly impaired colony formation in MaNRAS1014 and WM4265.2 cell lines, compared with either agent alone (Fig. 5A and B). Western blot analysis of WM4265.2, M93–047, and MaNRAS1014 cell lines treated with the combination of AP-4–139B and PD901 or trametinib led to increased markers of apoptosis (cleaved lamin A and cleaved caspase-3) compared with either agent alone (Fig. 5C and D). We next sought to test this drug combination *in vivo*.

**FIGURE 6:**
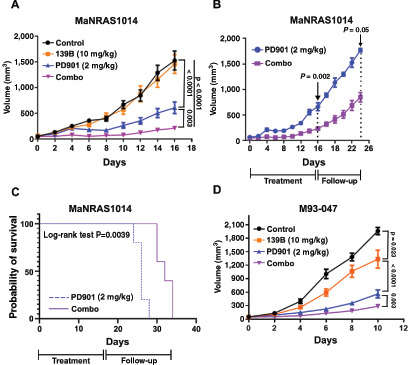
The HSP70 inhibitor AP-4–139B significantly enhances the durability of treatment of *NRAS*-mutant melanoma with PD901. **A**–**C,** 2.5 × 10^6^ MaNRAS1014 cells were subcutaneously injected into the flanks of NOD.Cg-PrkdcscidIl2rgtm1Wjl/SzJ (NSG) mice (*n* = 7–10 mice per group). Once tumors reached ∼50 mm^3^ mice were randomly assigned to each treatment group: vehicle, PD901 (2 mg/kg/day), AP-4–139B (10 mg/kg every two days), or combination (combo). Tumor growth was measured using digital calipers. The rate of tumor growth for each treatment group was calculated using a linear mixed model. **B,** MaNRAS1014 tumor rebound for PD901 and Combo treatment groups was assessed upon cessation of treatments (day 16, *n* = 5 mice per group). **C,** Kaplan–Meier survival curve of MaNRAS1014 tumor–bearing mice following cessation of treatment at day 16. Significance was determined using a log-rank test. **D,** 1 × 10^6^ M93–047 cells were subcutaneously injected into the flanks of NSG mice (*n* = 10–12 mice per group). Once tumors reached approximately 50 mm^3^ mice were randomly assigned to the indicated treatment groups. Tumor growth was measured using digital calipers, and the rate of tumor growth was measured using a linear mixed model.

We subcutaneously injected cells from the MaNRAS1014 or M93–047 *NRAS*-mutant melanoma cell lines into the flanks of NSG mice. When tumors reached approximately 50 mm^3^, mice were divided into four groups (*n* = 7–12/group): vehicle, AP-4–139B (i.p., 10 mg/kg every other day), PD901 (2 mg/kg/day oral gavage), and combination (AP-4–139B 10 mg/kg every other day; PD901 2 mg/kg/day). Analysis of tumor growth velocity revealed significant efficacy of PD901 as a single agent against MaNRAS1014 tumors, along with markedly improved efficacy of the combination, as evident by the significant decrease in tumor velocity with the combination therapy (*P* = 0.003, combination vs. PD901 alone, Fig. 6A). For MaNRAS1014 tumors, we ended the treatment and allowed tumors to rebound; again, the combination therapy led to significantly decreased velocity of tumor rebound (*P* < 0.05) and significantly improved survival (*P* < 0.01, Fig. 6B and C). In a second melanoma model of human M93–047 xenografts, the combination also provided significant benefit over each single agent (*P* < 0.003, Fig. 6D). The drug combination was well tolerated and there was no evidence of weight loss in combination-treated mice (Supplementary Fig. S5A and S5B).

## Discussion

Astrocytes interact with and protect brain metastases, including melanoma brain metastases, from many anticancer therapies; they can confer this protection through both contact-dependent and contact-independent (secretion) mechanisms (36). To date no groups have reported that astrocytes can increase the sensitivity of melanoma to therapy. We were therefore surprised to find that astrocyte coculture or conditioned media can sensitize melanoma tumor lines to MEKi. These data suggest that MEKi, and potentially also the BRAF/MEKi combination, might show enhanced efficacy in melanoma brain metastases. However, in general, MAPK inhibitors have shown poorer response rates for intracranial metastases compared with extracranial ones (37). To date, it has been unclear whether this is due to protection afforded from the brain microenvironment, or due to physical constraints, such as impaired ability of inhibitors to penetrate the blood–brain barrier and/or diffuse into tumors (38, 39). Our data support the latter possibility, and they suggest that BRAFi or MEKi with improved brain distribution could have significant benefit against brain metastases. In support of this premise, the recently developed MEKi E6201 displays improved brain distribution and shows potential promise against melanoma brain metastases (40, 41). A key unresolved issue in this article lies in the identification of the constituent in ACM that causes the increase in SREBP activity and the downregulation of ID3. Our data suggest that the increased SREBP activity may be responsible for the downregulation of ID3 by ACM (Supplementary Fig. S2E). However, we have been unable to identify the component of ACM that is responsible for upregulation of SREBP activity or the impact on MEKi sensitivity. Our add-back experiments in which ACM is supplemented with glucose or glutamine had no effect on the IC_50_ for MEKi, suggesting that the deprivation of these nutrients is unlikely playing a role. Whether the increased SREBP activity induced by astrocytes is caused by a secreted protein, a miRNA, or altered levels of cholesterol remains to be determined.

Our study identified the gene encoding the transcriptional regulator ID3 as one that is upregulated by MEKi in multiple melanoma cell lines. ID3 is a member of the inhibitor of differentiation (ID) family of proteins whose canonical function is to prevent the interaction of basic helix–loop–helix (bHLH) transcription factors with DNA (42). ID proteins have been studied for their ability to direct neural development and promote tumorigenesis (34, 43–45). Overexpression of ID family members has been observed in multiple tumor types and can be associated with poor prognosis (44). Recently, ID3 was found to be upregulated in response to BRAFi, and to play a role in resistance to BRAF inhibition (37, 38). ID3 expression in normal tissues is typically low or undetectable, making this protein an attractive therapeutic target in cancer. In support of this, targeting the ID1/ID3 interaction with the E47 bHLH transcription factor is effective against breast and ovarian cancers (45, 46). In addition, targeting ID1 and ID3 protein expression via inhibition of TGFβ receptor led to reduced initiation and growth of glioblastoma multiforme tumors *in vivo* (47). However, to date, there are no small-molecule inhibitors for ID3. In this study, we show for the first time that ID3 is a client protein of HSP70, and that inhibition of HSP70 with our novel inhibitor, AP-4–139B, causes ID3 to misfold and accumulate in a detergent insoluble compartment in the cell. In the past decade, targeted therapies have allowed patients diagnosed with melanoma to experience significantly improved progression-free and overall survival. However, combating acquired and adaptive resistance to targeted therapies has been a challenge. Our data indicate that targeting ID3 with an HSP70 inhibitor can greatly improve the efficacy of MEKi. Our combined data support a model whereby inhibition of MEK causes upregulation of ID3, and that the activation of SREBP activity by astrocytes may prevent this upregulation. Similarly, through a client–chaperone interaction, HSP70 inhibitors can also prevent the upregulation of ID3, by causing insolubility of this protein. One issue not resolved is the importance of ID3 to the efficacy of our HSP70 inhibitor. Our data indicate that silencing ID3 leads to a 3- to 10-fold increase in cytotoxicity (decrease in IC_50_) for PD901 and trametinib (Fig. 3G; Supplementary Fig. S3D). These increases in cytotoxicity are very similar to what we find for combinations of AP-4–139B with PD901 or Trametinib (Fig. 5A and B, graphs in right panel). These data suggest that, at least with regard to the ability of HSP70i to enhance the efficacy of MEKi in *NRAS*-mutant melanoma, ID3 may be a critical client protein of HSP70. This remains to be formally determined.

*NRAS* is the second most common mutated oncogenic driver in melanoma, after BRAF mutations. While MEKi have shown clinical efficacy, their efficacy as single agents against *NRAS*-mutant melanoma has been modest (48). As such, combinations therapies have been sought for *NRAS*-mutant melanoma. Some preclinical studies have supported combining MAPK pathway inhibitors with agents that target nononcogene addiction for example using inhibitors of the HSP70 and HSP90 family. Like MEKi, HSP90 inhibitors have not shown promise as monotherapies, but these inhibitors synergize with chemotherapy and targeted therapies in preclinical and early-phase clinical studies, thus supporting the use of this combination (49). Notably, the HSP70 inhibitor we used here-in has been shown to enhance the immune response to melanoma tumors (35), thus supporting the combination of this compound with immune checkpoint inhibitors as well. Along these lines, other members of the HSP70 family like the mitochondrial-localized member GRP75 (HSPA9) are also emerging targets for melanoma (50). How inhibition of GRP75 and our mitochondrial-targeted HSP70 inhibitor differs in the therapy of melanoma remains an area of active investigation.

## Supplementary Material

Supplementary Data Supplementary materials and methods and figure legends.

Supplementary Figure S1 This figure shows increased resistance of WM4265.2 cells to cisplatin and doxorubicin after culturing in astrocyte conditioned media, increased sensitivity of WM4265.2 and WM983B cells to PD901 or PLX4720 (WM983B) after culturing in astrocyte conditioned media, and the gating strategy for cell cycle analysis of WM4265.2 and astrocyte co-culture.

Supplementary Figure S2 This figure shows enriched pathways and regulators in WM4265.2 cells in astrocyte conditioned media versus astrocyte media, confirmatory RT-qPCR in WM4265.2 and WM983B cells from RNA sequencing experiment, knockdown efficiency of siRNA targeting SREBF1 and resulting effects on WM4265.2 and WM983B sensitivity to PD901, and RT-qPCR of genes affected by U1866a treatment in WM4265.2 cells.

Supplementary Figure S3 This figure shows knockdown efficiency of siRNA targeting ID3 in WM983B and 1205Lu cells, proliferation curve of M93-047 cells containing shRNA targeting ID3, Western blot of lysates from M93-047 cells with and without shRNA targeting ID3, IC50 analysis of M93-047 with or without shRNA targeting ID3, trypan blue exclusion assay in M93-047 treated with PD901 with or without siRNA targeting ID3 and lysates from both M93-047 and WM4265.2 cells with or without siRNA targeting ID3 in the presence or absence of PD901 treatment.

Supplementary Figure S4 This figure shows interaction indices for MaNRAS1014 and WM4265.2 cells treated with AP-4-139B and PD901 or Trametinib combinations and cell cycle analysis of WM4265.2 and M93-047 cells treated with AP-4-139B, PD901 or the combination of AP-4-139B and PD901.

Supplementary Figure S5 This figure shows the change in weight of tumor bearing mice treated with the indicated compounds during the treatment period.
