# Supplementary material for: HSP70 Inhibition Blocks Adaptive Resistance and Synergizes with MEK Inhibition for the Treatment of NRAS-Mutant Melanoma
Source: Cancer Res Commun. 2021 Oct 13;1(1):17–29. doi: 10.1158/2767-9764.CRC-21-0033 (PMC8849551; doi:10.1158/2767-9764.CRC-21-0033)
Supplement: Supplementary Data — Supplementary materials and methods and figure legends. [file crc-21-0033-s01.docx]

**Supplemental Materials and Methods**

| **shRNA #** | **Sense Sequence** | **Full Hairpin** |
| --- | --- | --- |
| sh1 | GCCCACTTGACTTCACCAAAT | CCGGGCCCACTTGACTTCACCAAATCTCGAGATTTGGTGAAGTCAAGTGGGCTTTTT |
| sh2 | CATCGACTACATTCTCGACCT | CCGGCATCGACTACATTCTCGACCTCTCGAGAGGTCGAGAATGTAGTCGATGTTTTT |
| sh3 | TCAGCTTAGCCAGGTGGAAAT | CCGGTCAGCTTAGCCAGGTGGAAATCTCGAGATTTCCACCTGGCTAAGCTGATTTTT |
| sh4 | CGCTGAGCTTGCTGGACGACA | CCGGCGCTGAGCTTGCTGGACGACACTCGAGTGTCGTCCAGCAAGCTCAGCGTTTTT |

**ID3 Short Hairpin Sequences**

**Human qPCR Primer Sequences**

| **Gene Symbol** | **Forward Primer** | **Reverse Primer** |
| --- | --- | --- |
| *ID3* | TCAGGAGCGAAGGACTGTGA | CCGCCTTGGCATAGTTTGGA |
| *SREBF1* | CTGACCGACATCGAAGGTGA | AAGTGCAATCCATGGCTCCG |
| *ACCβ* | TACCGTCACTTGGAACCTGC | ATGGTCCGTCACTTCCACAC |
| *FASN* | CCCCTGATGAAGAAGGATCA | ACTCCACAGGTGGGAACAAG |
| *SREBF2* | CGCCTCCTAGGATGTTAGCC | GTGGAGGTAGGAGATGGGG |
| *ACSS2* | CAAGGGTGTGGTTCACACAG | GCCCATAGGTGACGTAGGAA |
| *HMGCR* | GGCCCAGTTGTGCGTCTT | CGAGCCAGGCTTTCACTTCT |
| *GAPDH* | GACAGTCAGCCGCATCTTCT | GCGCCCAATACGACCAAATC |
| *TBP* | GTTCCAGCGCAAGGGTTTCT | GTAAGGTGGCAGGCTGTTGT |

**Supplemental Figure legends**

**Supplemental Figure 1:** (**A**) Cell viability of WM4265.2 cultured in AM or ACM and treated with cisplatin or doxorubicin for 72 hours. Cell viability was assessed using alamarBlue assays, n=3. ***­ p-value < 0.001 as per two-tailed student’s t-test. (**B**) IC_50_ of WM4265.2 and WM983B cell lines incubated in AM or ACM and treated with PD901 or the BRAF inhibitor PLX4720 for 72 hours, n=6. (**C**) Gating strategy for WM4265.2 mono- and WM4265.2 astrocyte co-cultures. GFP+ WM4265.2 cells from mono- or astrocyte co-cultures (top panels) were gated for cell cycle analysis using propidium iodide (bottom).

**Supplemental Figure 2:** (**A**) Ingenuity pathway analysis (IPA) showing top affected cellular pathways in WM4265.2 cells RNA-sequencing experiment. Pathways shown were overrepresented in ACM compared to AM. (**B**) IPA regulator analysis showing top affected transcriptional regulators in WM4265.2 cells RNA-sequencing experiment. Regulators with a Z-score > 2 were denoted as activated in ACM compared to AM. (**C**) Confirmatory RT-qPCR of the SREBP1/2 target *ACSS2* confirms increased SREBP1/2 activity in ACM treated cells, n=3. (**D**) Cell viability (left) of WM4265.2 and WM983B cell lines with siRNA targeting *SREBF1* (right) and treated with PD901­­­ for 72 hours. Cell viability was assessed using alamarBlue assays, n=6. RT-qPCR of *SREBF1* in WM4265 and WM983B cell lines incubated with siRNA targeting *SREBF1* or non-targeting control (si-Ctrl), n=3. (**E**) RT-qPCR of SREBP1/2 target genes in WM4265.2 cells treated with 2.5 µM U1866a for indicated time points. *** p-value < 0.001, ** p-value < 0.01, * p-value < 0.05; assessed by two tailed student’s t-test. Note down-regulation of ID3 in cells treated with the SREBP1/2 agonist.

**Supplemental Figure 3:** (**A**) WM983B (left) and 1205Lu (right) cell lines transfected with siRNA targeting ID3 (si-ID3) or non-targeting control (si-Ctrl) for 24 hours and analyzed for ID3 level by RT-qPCR, n=3. (**B**) Cell counts of M93-047 cells stably-infected with two independent shRNA constructs targeting ID3 (shID3_1 and shID3_4). Data shown are the mean +/- standard deviation, n=3. (**C**) Western blot probed with indicated antibodies of lysates from M93-047 cells with shRNA targeting ID3 and treated with 1 µM PD901 for 24 hours. (**D**) IC_50_ analysis of M93-047 cells with stable knockdown of ID3 treated with PD901 or Trametinib for 72 hours. Cell viability was assessed using alamarBlue assays, n=6. (**E**) Trypan blue exclusion analysis of cell viability in M93-047 cells transfected with siRNA targeting ID3 or control siRNA, followed by treatment with 1 µM PD901 for 72 hours, n=3. * = p < 0.05, assessed by two-tailed student’s t-test. (**F**) Western blot for ID3 and cleaved lamin A (apoptosis) of lysates from M93-047 cells transfected with siRNA targeting ID3 or control siRNA, followed by treatment with 1 µM PD901 for 72 hours. (**G**) Western blot probed with indicated antibodies of lysates from WM4265.2 cells with siRNA knockdown of ID3 and treated with 1 µM PD901 for 24 hours.

**Supplemental Figure 4:** Synergy of AP-4-139B and MEKi in MaNRAS1014 and WM4265.2 cells. (**A-B**) Contour plot (left) and interaction index (right) assessing synergy of combination of PD901 and AP-4-139B (**A**) and Trametinib and AP-4-139B (**B**) in MaNRAS1014 cells. MaNRAS1014 cells were treated for 72 hours, and cell viability was analyzed using CellTiterGlo. (**C**) Contour plot (left) and interaction index (right) showing synergy of PD901 and AP-4-139B (**C**) and Trametinib and AP-4-139B (**D**) in WM4265.2 cells. WM4265.2 cells were treated for 72 hours, and cell viability was analyzed using CellTiterGlo. Interaction index between PD091, Trametinib and AP-4-139B was determined using the Bliss independence model. Interaction index <1 is indicative of synergy at indicated doses. (**E**) Cell Cycle analysis of WM4265 (left) and M93-047 (right) cells treated with 1 µM PD901, 5 µM AP-4-139B, or the combination for 24 hours. Data represent the mean +/- standard deviation, n=3. *** = p < 0.001, ** = p < 0.01, as assessed by One-way Anova.

**Supplemental Figure 5:** (**A**) Weight (grams) of MaNRAS1014 tumor-bearing mice throughout treatment (n = 7-10 per group). Mice were weighed every two days. (**B**) Weight (grams) of M93-047 tumor bearing mice throughout duration of treatment (n = 10-12 mice per group). Mice were weighed every two days.
