# Supplementary figures and images for: HSP70 Inhibition Blocks Adaptive Resistance and Synergizes with MEK Inhibition for the Treatment of NRAS-Mutant Melanoma
Source: Cancer Res Commun. 2021 Oct 13;1(1):17–29. doi: 10.1158/2767-9764.CRC-21-0033 (PMC8849551; doi:10.1158/2767-9764.CRC-21-0033)

Supplemental Figure S1

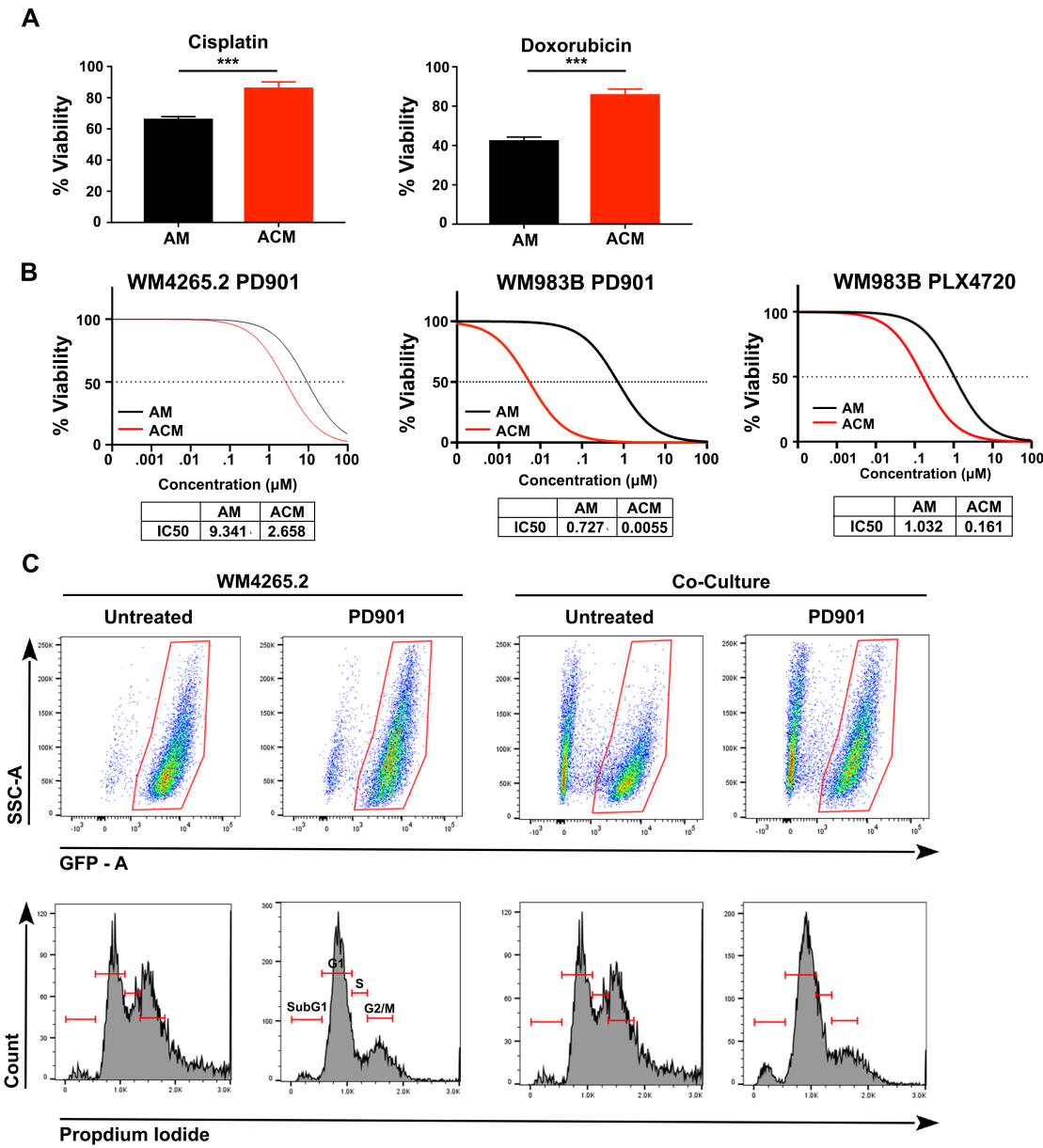

Supplement: Supplementary Figure S1 — This figure shows increased resistance of WM4265.2 cells to cisplatin and doxorubicin after culturing in astrocyte conditioned media, increased sensitivity of WM4265.2 and WM983B cells to PD901 or PLX4720 (WM983B) after culturing in astrocyte conditioned media, and the gating strategy for cell cycle analysis of WM4265.2 and astrocyte co-culture. [file crc-21-0033-s02.pdf]

Supplemental Figure S2

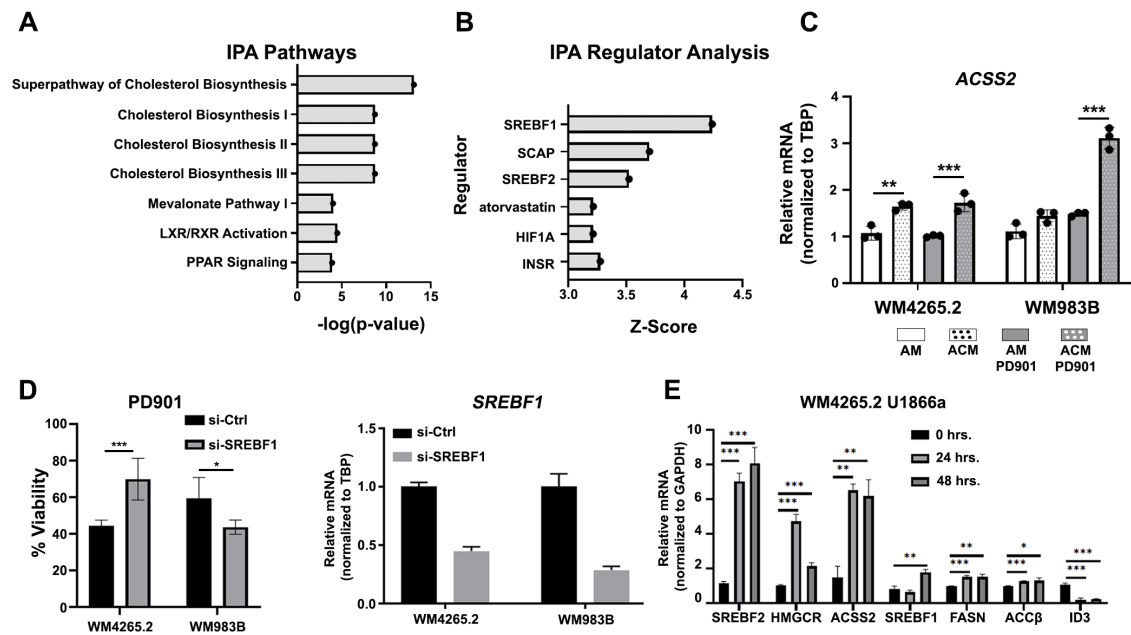

Supplement: Supplementary Figure S2 — This figure shows enriched pathways and regulators in WM4265.2 cells in astrocyte conditioned media versus astrocyte media, confirmatory RT-qPCR in WM4265.2 and WM983B cells from RNA sequencing experiment, knockdown efficiency of siRNA targeting SREBF1 and resulting effects on WM4265.2 and WM983B sensitivity to PD901, and RT-qPCR of genes affected by U1866a treatment in WM4265.2 cells. [file crc-21-0033-s03.pdf]

Supplemental Figure S3

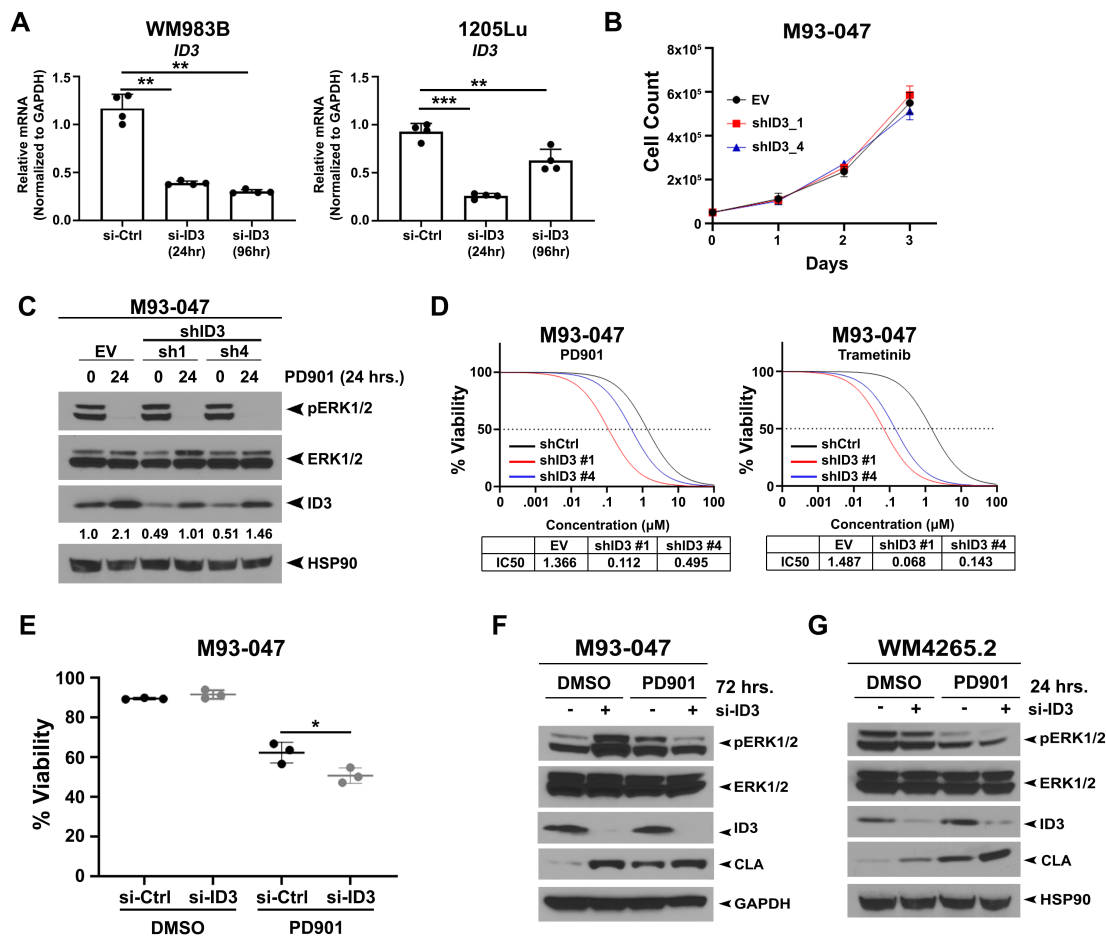

Supplement: Supplementary Figure S3 — This figure shows knockdown efficiency of siRNA targeting ID3 in WM983B and 1205Lu cells, proliferation curve of M93-047 cells containing shRNA targeting ID3, Western blot of lysates from M93-047 cells with and without shRNA targeting ID3, IC50 analysis of M93-047 with or without shRNA targeting ID3, trypan blue exclusion assay in M93-047 treated with PD901 with or without siRNA targeting ID3 and lysates from both M93-047 and WM4265.2 cells with or without siRNA targeting ID3 in the presence or absence of PD901 treatment. [file crc-21-0033-s04.pdf]

Supplemental Figure S4

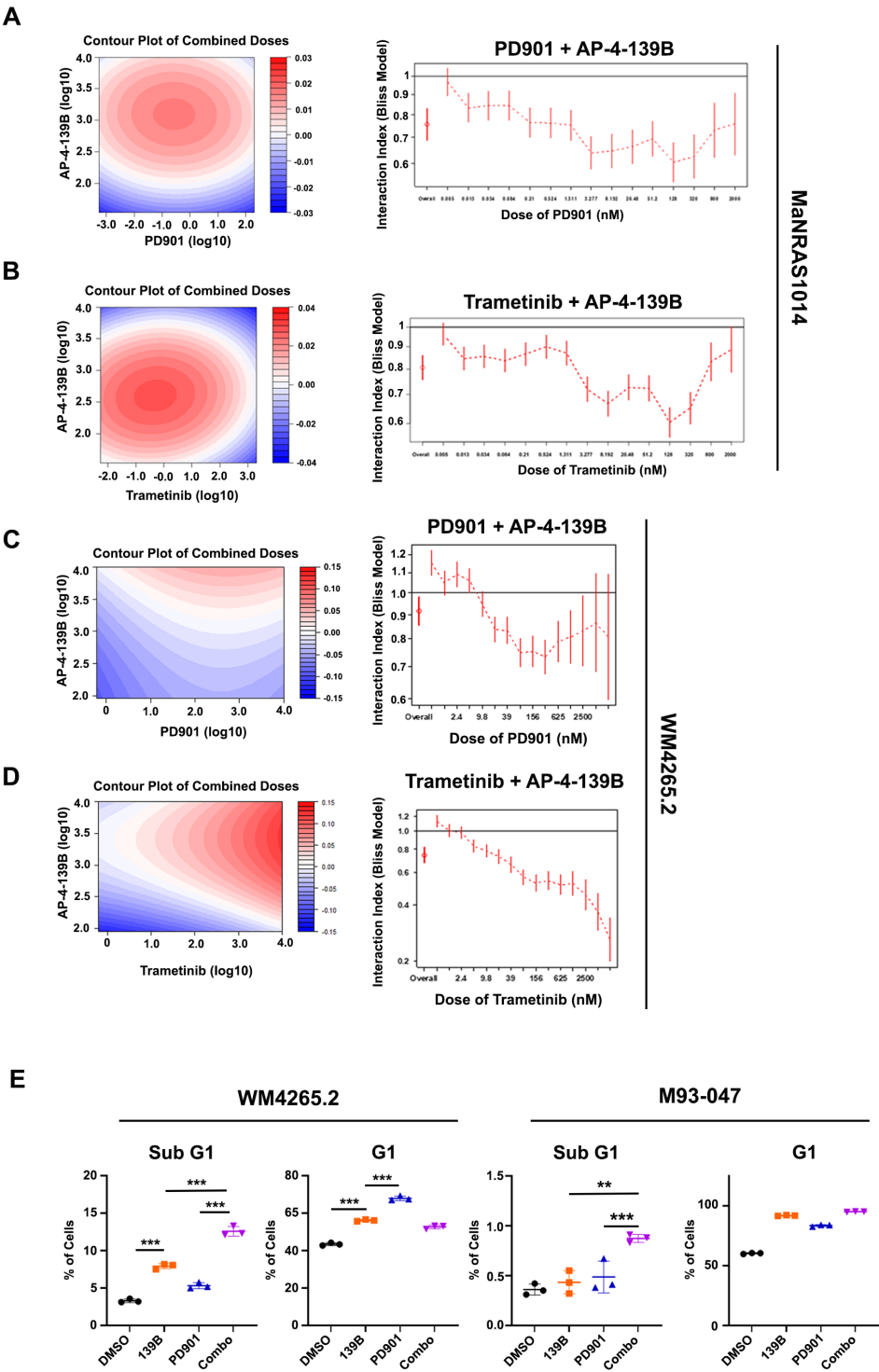

Supplement: Supplementary Figure S4 — This figure shows interaction indices for MaNRAS1014 and WM4265.2 cells treated with AP-4-139B and PD901 or Trametinib combinations and cell cycle analysis of WM4265.2 and M93-047 cells treated with AP-4-139B, PD901 or the combination of AP-4-139B and PD901. [file crc-21-0033-s05.pdf]

Supplemental Figure S5

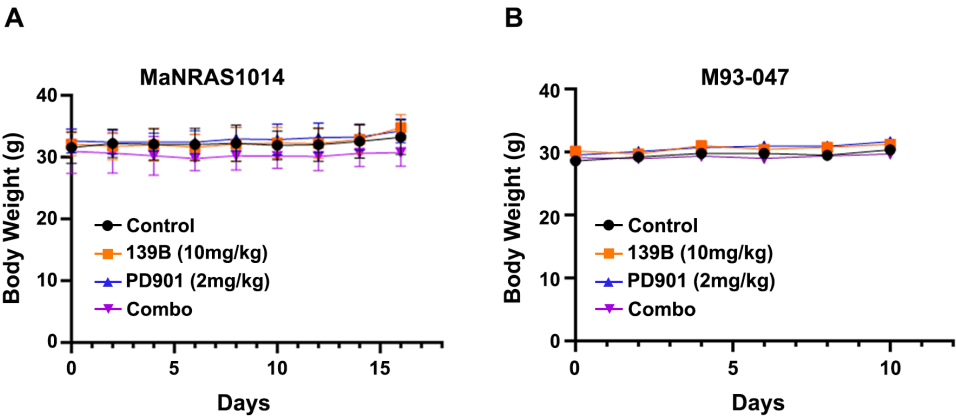

Supplement: Supplementary Figure S5 — This figure shows the change in weight of tumor bearing mice treated with the indicated compounds during the treatment period. [file crc-21-0033-s06.pdf]
